# Noninvasive direct bilirubin detection by spectral analysis of color images using a Mini-LED light source

**DOI:** 10.1186/s11671-023-03794-9

**Published:** 2023-02-16

**Authors:** Hsin-Ching Kuo, Zhi-Ting Ye, Shen Fu Tseng, Shang Xuan Tsou, Shih Wei Huang, Chun-Wei Tsai

**Affiliations:** 1 Ditmanson Medical Foundation Chia-Yi Christian Hospital, Chiayi City, Taiwan, ROC; 2 Department of Mechanical Engineering, Advanced Institute of Manufacturing with High-Tech Innovations, National Chung Cheng University, 168, University Rd., Min-Hsiung, Chia-Yi, 62102 Taiwan, ROC; 3 Department of Engineering, i-Wavefront Technology Ltd., 6F.-5, NO. 95, Minquan Rd., Xindian Dist., New Taipei City, 231625 Taiwan, ROC

**Keywords:** Direct bilirubin, Test paper, Linear relationship, Quantitative detection, Spectral, Mini-LEDs

## Abstract

Urine test paper is a standard, noninvasive detection method for direct bilirubin, but this method can only achieve qualitative analysis and cannot achieve quantitative analysis. This study used Mini-LEDs as the light source, and direct bilirubin was oxidized to biliverdin by an enzymatic method with ferric chloride (FeCl_3_) for labeling. Images were captured with a smartphone and evaluated for red (R), green (G), and blue (B) colors to analyze the linear relationship between the spectral change of the test paper image and the direct bilirubin concentration. This method achieved noninvasive detection of bilirubin. The experimental results demonstrated that Mini-LEDs can be used as the light source to analyze the grayscale value of the image RGB. For the direct bilirubin concentration range of 0.1–2 mg/dL, the green channel had the highest coefficient of determination coefficient (*R*^2^) of 0.9313 and a limit of detection of 0.56 mg/dL. With this method, direct bilirubin concentrations higher than 1.86 mg/dL can be quantitatively analyzed with the advantage of rapid and noninvasive detection.

## Introduction

Bilirubin is a yellow pigment produced by the catabolism of hemoglobin in red blood cells, and it is eventually metabolized by the liver to be excreted from the body [1–3]. Bilirubin in human serum is mainly classified according to whether or not it is esterified by enzymes. Bilirubin that is not bound to other substances such as glucuronic acid is called indirect bilirubin or free bilirubin. Indirect bilirubin binds to albumin and is transported in the plasma to the liver for metabolism. Once it reaches the liver, it is combined with other substances, such as esterified compounds and glucuronic acid in liver cells, where it is then called conjugated bilirubin or direct bilirubin. These two types of bilirubin are collectively called total bilirubin [4]. Since the kidneys only filter direct bilirubin and excrete it into the urine, and indirect bilirubin is insoluble in water, the values obtained are mostly direct bilirubin. High concentrations of total bilirubin in the body can cause skin and other tissue lesions and yellow discoloration, resulting in a condition called jaundice or hyperbilirubinemia [5].

Medically, the jaundice disease of newborns is called neonatal jaundice. The rate of neonatal jaundice is relatively high, at about 60%, especially higher in premature babies. Therefore, bilirubin tests are routine for every newborn [6–8]. Neonatal jaundice symptoms are divided into physiological jaundice and pathological jaundice. Physiological neonatal jaundice is mainly caused by the poor metabolic function of neonatal livers and will gradually subside due to the improvement in neonatal metabolic function over time [9–11]. Most cases of physiological neonatal jaundice do not cause serious sequelae, but abnormally high values of bilirubin can potentially lead to neurotoxicity, neurodevelopmental abnormalities such as hearing loss, variable spasms, athetosis, and even mental deficits [12]. There are two main reasons for the occurrence of pathological jaundice. One is biliary atresia, and the other is neonatal hepatitis. The symptoms of biliary atresia require early diagnosis and surgical intervention to prevent the symptoms from worsening and eventually necessitating liver replacement [13, 14]. When neonatal jaundice lasts for more than 2 weeks, it is clinically called delayed jaundice. At this time, further examination is required to confirm whether there is a possibility for pathological jaundice.

Currently, the bilirubin detection methods used in hospital laboratories are primarily oxidase and diazo methods [15, 16]. In the diazo method, bilirubin and diazonium ions are coupled in a strong acid environment to form a dark red azo compound. A photometer is then used to measure the fluorescence intensity and compares it with the ratio of bilirubin concentration color [17]. Edachana et al. proposed a direct bilirubin diazo detection method for urine samples by dropping chloroauric acid into bilirubin. This method utilizes the reduction of gold (III) ions by bilirubin and the formation of gold nanoparticles resulting in a color change from yellow to purple with an optimal absorption peak at 530 nm. It functions in the bilirubin concentration range of 5.0 to 1000 mg/mL with a detection limit of 1.0 mg/mL and a sample recovery rate of over 95% [18]. Lano et al. proposed the use of co-oximetry (Radiometer (R) ABL90) to analyze total bilirubin concentration. The samples were centrifuged and analyzed for total bilirubin in plasma with diazonium reagents. The mean deviation over the range of total bilirubin levels was − 1.0 μmol/L [19]. Although the diazo method of bilirubin detection is inexpensive and easy to automate, it is easily affected by other substances and the pH value of the environment, making the measured bilirubin concentration inaccurate and increasing the overall detection time [20–22]. Bilirubin oxidase (BOD) is a multi-copper oxidase belonging to the class of oxidoreductases. It uses metal ions to catalyze the oxidation of bilirubin to biliverdin and has been widely used as a reagent for testing bilirubin [23]. Rawal et al. proposed immobilizing BOD on graphene oxide nanoparticles (GrONPs) to fabricate a bilirubin biosensor. For a bilirubin concentration range of 0.01 to 600 μM, it had a correlation coefficient *r* of 0.9939 [24]. Zhang et al. proposed the development of *Bacillus subtilis* pore coat protein A (CotA) to measure bilirubin. CotA exhibits a specific oxidative capacity for direct bilirubin in an acidic environment and total bilirubin in an alkaline environment. The appropriate pH conditions for CotA to detect direct bilirubin and total bilirubin are 5.5 and 7.5, respectively [25]. Batra et al. proposed to covalently immobilize BOx on zirconia-coated silica nanoparticles (SiO¬2@ZrONPs)/chitosan (CHIT) composites. In the concentration range of 0.02 to 250 μM, the method had a detection limit of 0.1 nM and bilirubin recoveries of 95.56–97.0% [26]. The method of using oxidase to detect bilirubin is widely used in testing reagents. However, two disadvantages of this strategy are that oxidases are easily interfered with by other substances, and the enzyme must be separated in an aqueous solvent [27–29]. Thus, oxidase can only be detected qualitatively at present, and there is still a need to develop an accurate method for quantitative detection and analysis.

Blood testing is currently the most accurate method for detecting human bilirubin, but this method is invasive, increases the risk of infection for certain patients, and involves a higher cost of labor. In addition, the bilirubin value can only be correctly interpreted through a specific detection instrument making it relatively difficult for the patient to perform the test [30]. However, the cost of testing equipment is costly. Parnianchi et al. proposed a noninvasive electrochemical sensor to detect bilirubin in the saliva [31]. The detection of bilirubin in urine using test papers is another noninvasive testing method [32]. However, most test papers require visually detecting a color change as the basis for bilirubin detection. If the bilirubin concentration is low or the quality of the test paper is poor, the color change will not be obvious, and the detection by the user may be inaccurate.

In optical biomedical testing, a halogen (H2) lamp is often used as the light source of visible light to near-infrared wavelengths [33–36]. Mini-LEDs and Micro-LEDs have the advantages of high brightness, long life, high color purity, and high efficiency [37, 38].

Blue Mini-LEDs is used as a light source to designed a miniaturized optomechanical device for the detection of direct bilirubin [39]. Ye et al. proposed using blue Mini-LEDs as a light source to detect direct bilirubin, in the concentration range from 0.855 to 17.1 μmol/L the *R*^2^ was 0.9999 [39]. In addition, smartphone cameras have proven to be excellent analytical devices for digitizing images to visualize data in recent years, since they are the most popular and easy-to-use scanners with high sensitivity to light and color variations through RGB analysis apps or software [40, 41]. Smartphones offer the advantages of cost-effectiveness, portability, and ease of operation for analyte detection through RGB analysis apps or software [42, 43]. Xu et al. proposed using spotlight LEDs as a light source and capturing the picture by smartphone to detect bilirubin in whole blood it has the advantages of significant portability, low cost, instrument-free, and high sensitivity [44]. Tabatabaee et al. proposed using blue light LEDs as a light source and capturing the picture by smartphone to detect bilirubin in whole blood. The recovered PL intensity has linearly proportional to the concentration of bilirubin in the range of 2–20 mg/dL [45]. Previous studies have yet to develop noninvasive bilirubin detection by color image analysis. This study proposes noninvasive direct bilirubin detection by spectral analysis of color images using Mini-LEDs as a light source. Images were captured by a smartphone and then analyzed using the relationship of the direct bilirubin test paper with different concentrations corresponding to the average RGB grayscale to achieve the goal of noninvasive bilirubin test papers. This method has the advantages of being noninvasive, rapid, and portable.

## Materials and methods

### Linear regression analysis

The coefficient of determination (*R*^2^) is defined as the regression equation variation value and all variations in the proportion of the quantity. The value of *R*^2^ is represented by 0–1, and the larger the value of *R*^2^, the better the regression equation can explain the overall variation. The coefficient of determination *R*^2^ is shown in Eq. 1:

1 $$R^{2} = \sum (\widehat{{Y_{i} }} - \overline{Y})^{2} /\sum (Y_{i} - \overline{Y})^{2}$$

where $\widehat{{Y_{i} }}$ is the predicted value of the regression model at point $Y_{i} { }$, $\overline{Y}$ is the average value of all $Y_{i}$ values, and *R*^2^ represents the ratio of the variance value of the regression model to all the *Y* variances.

### Experimental materials

Direct bilirubin powder (Bilirubin Conjugate, Ditaurate, Disodium Salt—Calbio-chem, Merck Millipore 201102, Inc, USA) is the standard material currently used by medical institutions to simulate direct bilirubin for detection. The certificate of analysis (CoA) according to Regulation (EC) No.1907/2006 of direct bilirubin powder, and the safety data sheet (SDS) of direct bilirubin powder is shown in Table 1.

**Table 1:** The safety data sheet (SDS) for direct bilirubin

	Matter	Information
1	Description	Suitable as a direct bilirubin standard
2	Inert gas (yes/no)	Packaged under inert gas
3	Registry of Toxic Effects of Chemical Substances (RTECS)	DU3038000
4	Solubility	H_2_O (10 mg/mL)
5	Storage	Protect from light − 20 °C Hygroscopic

**Fig. 1:**
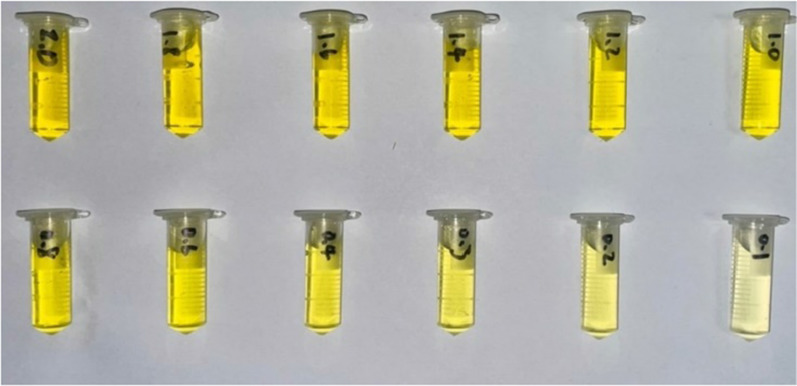
The direct bilirubin samples with concentrations of 0.1–2.0 mg/dL

The preparation procedure was to add 25 mL of deionized water to 0.5 mg of direct bilirubin powder to form a solution of 2 mg/dL. Next, deionized water was added to dilute to 12 standard solutions of different concentrations. The concentration range was 0.1–2 mg/dL, as shown in Fig. 1.

The weight of direct bilirubin and deionized water was measured using a precision scale (IAXL-GR-120, A&D, Japan), and stirred for 5 min at 500 rpm by a magnetic stirrer to mix uniformly.

Fouchet's Reagent (S-Y Fouchet Reagent-SY8076-1-11011019-Shih-Yung Instruments Co., Ltd) was used as an oxidase. Fouchet's Reagent oxidizes direct bilirubin to biliverdin, which is blue-green, and the direct-type bilirubin is labeled with this reaction, as shown in Eq. 2:

2 $${\text{Biliverdin}} + {\text{FeCl}}_{3} \;\overrightarrow {{({\text{Fouche's solution}})}} \;{\text{Biliverdin}} + {\text{Fe}}^{3 + }$$

Direct bilirubin test paper (S-Y U-B Test Kit Lot No 1110412-SY8076-11011019-Shih-Yung Instruments Co., Taiwan Ltd) was used as the analytical direct bilirubin carrier, with a diameter of 30 mm.

### Color image spectral analysis

**Fig. 2:**
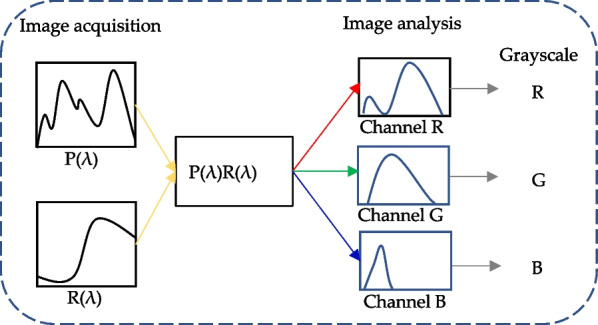
Schematic diagram of the direct detection of bilirubin by color image spectral analysis

The color image analysis proposed in this study is divided into two parts. The first part is the image acquisition of the test paper image. *P*(*λ*) and *R*(*λ*) are the light source spectrum distribution and reflection spectrum, respectively. By multiplying *P*(*λ*) and *R*(*λ*), the spectrum of direct bilirubin after the reaction on the test paper can be obtained. The second part is image analysis. MATLAB software was used to divide the RGB value of the image into three channels for numerical analysis: R, G, and B. The RGB grayscale value was divided by the same sampling pixel number, and the average grayscale value for each color group was used for the direct detection of bilirubin by color image spectral analysis, as shown in Fig. 2.

### Schematic diagram of experimental setup

**Fig. 3:**
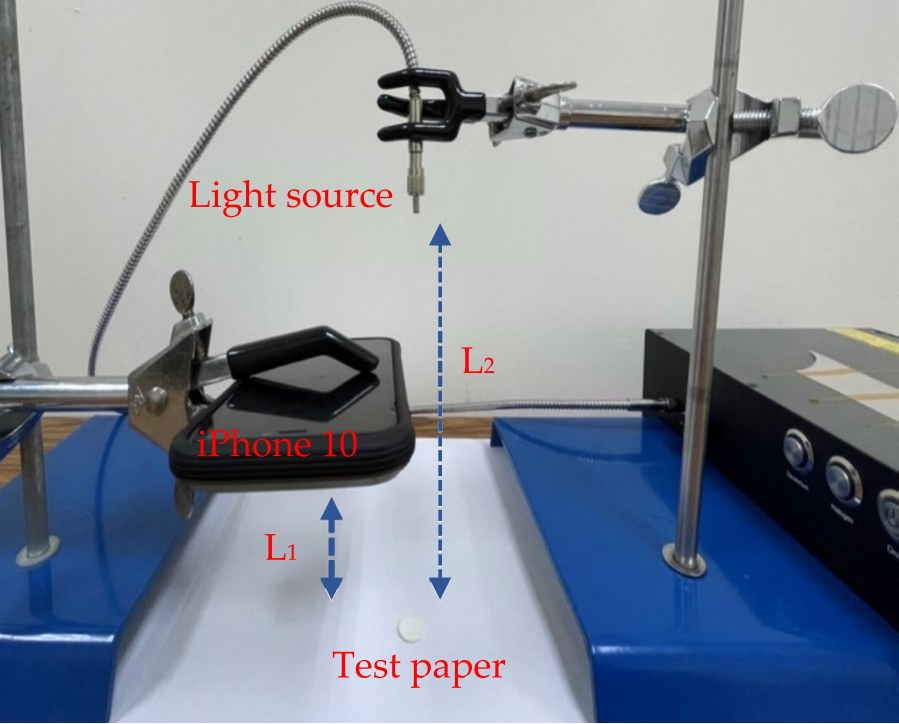
The experimental setup for direct bilirubin detection by spectral analysis of color images

The experimental setup is shown in Fig. 3. The test paper was fixed at 90 degrees to the lens for image acquisition. *L*_1_ is the distance between the camera lens and the test paper sample, and *L*_2_ is the distance between the light source and the test paper. *L*_1_ and *L*_2_ were 10 cm and 15 cm, respectively. The light source D2 lamp and H2 lamp were transmitted through a 1-m optical fiber, and Mini-LEDs were fixed directly above. Using an iPhone 10 camera to capture images with a resolution of 24 megapixels, 13,528 pixels were captured in the area of the test paper, with a diameter of 30 mm as the analysis range.

### Experimental process

**Fig. 4:**
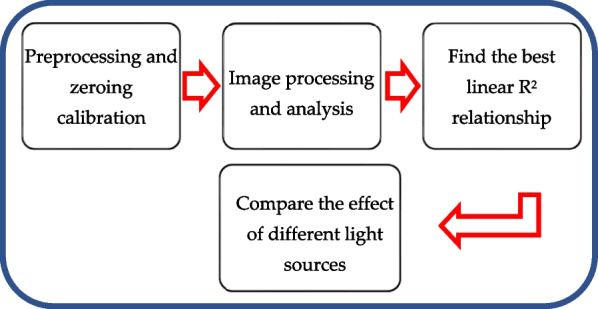
Experimental process of direct bilirubin test papers using image recognition and different light sources

In this study, a D2 lamp, H2 lamp, and Mini-LEDs were used as the light source to capture images with a smartphone in the darkroom, and the captured images were analyzed using MATLAB. The experimental process of detecting direct bilirubin using a test paper is shown in Fig. 4. Following pretreatment and zero calibration, 12 different concentrations of direct bilirubin standard solutions (0.1–2.0 mg/dL) were prepared, and 0.1 mL of Fouchet's Reagent was added to the solution as oxidase. The parameters such as the distance between the light source and the optical fiber and the focal length and sensitivity of the smartphone were adjusted. After the calibration of the measurement parameters was completed, the images of 12 direct bilirubin test papers with different concentrations were acquired. The test paper image RGBs were analyzed for the different concentrations based on its average grayscale value, and the linear relationship between the direct bilirubin concentration and the RGB average was determined using a binary linear equation and *R*^2^. Finally, the measured results of the deuterium lamp (D2 lamp), halogen lamp (H2 lamp), and Mini-LEDs were compared based on the linearity of the corresponding direct bilirubin.

## Results and discussion

**Fig. 5:**
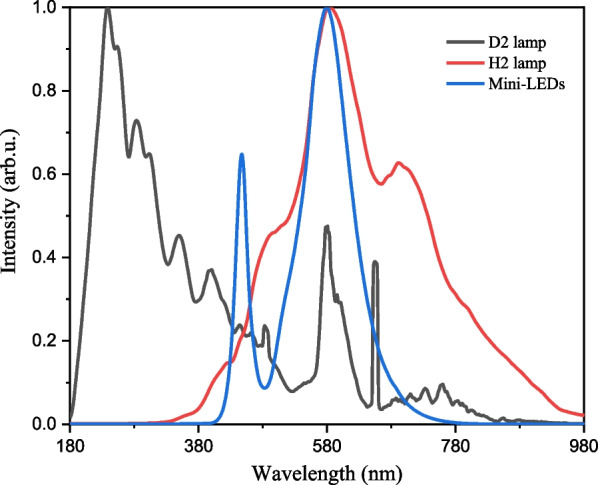
Emission spectra of D2 lamp, H2 lamp, and Mini-LEDs

In this study, three light sources were used for detection. Figure 5 shows the emission spectra of the deuterium lamp (D2 lamp), halogen lamp (H2 lamp), and Mini-LEDs light source, respectively. When the spectrum of the light source is concentrated and close to the absorbance wavelength of the analyte, the noise signal can be reduced.

### Image detection of bilirubin test paper using D2 lamp as the light source

**Fig. 6:**
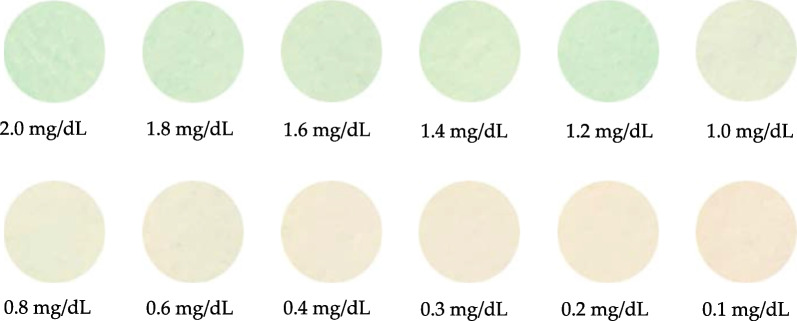
Color images of test papers with different concentrations of bilirubin while using a D2 lamp as the light source

First, a D2 lamp was used as the light source to detect direct bilirubin with a concentration range of 0.1–2.0 mg/dL. The direct bilirubin test paper sample captured by an iPhone 10 is shown in Fig. 6. It is evident from the sample photographs that when the concentration of direct bilirubin is higher, the color on the test paper is darker. This is because when the bilirubin concentration is high, the relative proportion of biliverdin increases, and the color on the test paper changes as the biliverdin deposited on the test paper increases.

The image processing was conducted with MATLAB by dividing the captured image into three channels (R, G, and B) for spectral analysis of the images. In the extracted 13,528 pixels, the average grayscale value and the direct bilirubin concentration were used for linearity analysis. The *R*^2^ of bilirubin test papers in the G, R, and B channels were 0.7809, 0.2555, and 0.0738, respectively.

**Fig. 7:**
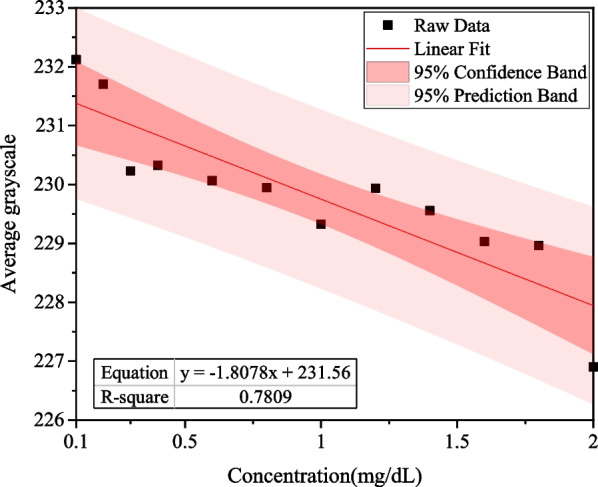
Linear relationship between different concentration of direct bilirubin test paper and average grayscale of G channel using a D2 lamp

The experimental results show that when a D2 lamp is used as the light source, the linear relationship for the green channel is the best. The linear equation of the G channel was *y* = − 1.*8078x* + 231.56, and *R*^2^ was 0.7809, as shown in Fig. 7.

### Image detection of bilirubin test paper using an H2 lamp as the light source

**Fig. 8:**
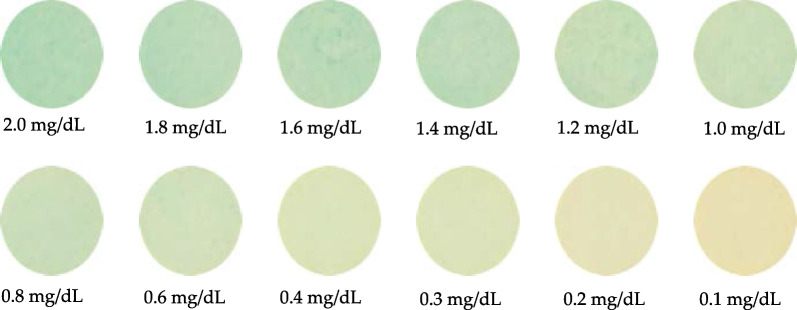
Color images of test papers with different concentrations of bilirubin while using an H2 lamp as the light source

Next, an H2 lamp was used as the light source, and the pictures for twelve different concentrations are shown in Fig. 8.

The *R*^2^ for bilirubin test papers in the G, R, and B channels were 0.8984, 0.8155, and 0.3064, respectively.

**Fig. 9:**
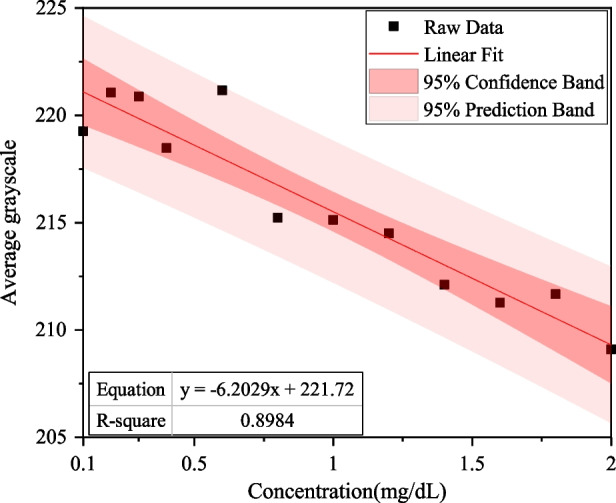
Linear relationship between different concentrations of direct bilirubin and average grayscale of the G channel while using an H2 lamp as the light source

The results show that when an H2 lamp is used, the linear relationship for the green channel is the best. The linear equation for the G channel was *y* = − 6.2029*x* + 221.72, *R*^2^ was 0.8984, and the LOD was 0.69 mg/dL, as shown in Fig. 9.

### Image detection of bilirubin test papers using Mini-LEDs as the light source

**Fig. 10:**
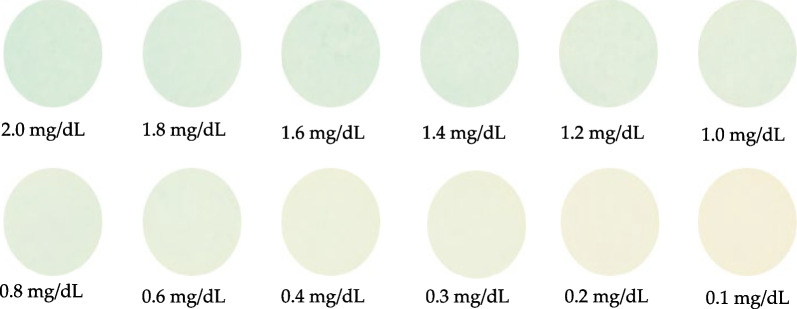
Color images of test papers with different bilirubin concentrations while using Mini-LEDs as the light source

Finally, Mini-LEDs were used as the light source to detect direct bilirubin via spectral analysis. The pictures of twelve different bilirubin concentrations are shown in Fig. 10.

The *R*^2^ of bilirubin test papers for the G, R, and B channels were 0.9313, 0.8522, and 0.2877, respectively.

**Fig. 11:**
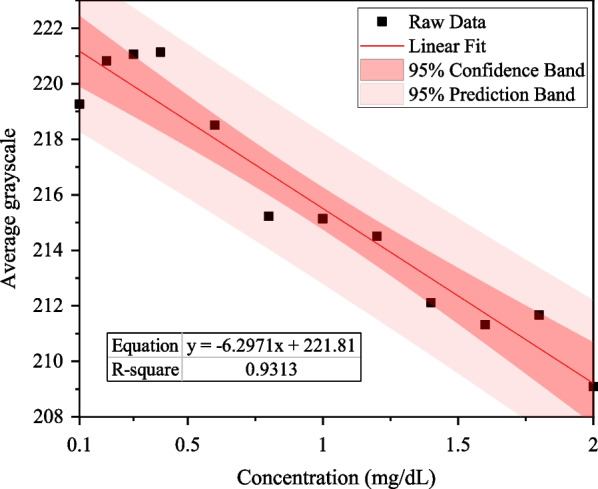
Linear relationship between different concentrations of direct bilirubin and average grayscale of the G channel while using Mini-LEDs as the light source

The results show that when Mini-LEDs were used, the linear relationship for the green channel was the best. The linear equation of the G channel was *y* = − 6.2971*x* + 221.81, *R*^2^ was 0.9313, and the LOD was 0.56 mg/dL, as shown in Fig. 11.

### Analysis and comparison of different light sources

This study proposes to measure direct bilirubin using test papers and D2, H2, and Mini-LEDs as the light source. The linear relationship between direct bilirubin and the average RGB grayscale values was analyzed for the concentration range of 0.1–2.0 mg/dL. As shown in Table 2, when a D2 lamp, H2 lamp, and Mini-LEDs were used as the light source, the linear relationship for the green channel was the best, and *R*^2^ values were 0.7809, 0.8984, and 0.9313, respectively. Since the spectrum of the Mini-LED is concentrated and close to the absorbance wavelength of the analyte, the noise signal can be reduced and has higher linearity compared with D2 and H2 lamp.

**Table 2:** Color images analysis for test paper using different light sources

Light source	D2 lamp	H2 lamp	Mini-LEDs
Peak wavelength	238.8 nm	583.8 nm	580.4 nm
Wavelength range	190–780 nm	380–930 nm	380–780 nm
Color coordinate (*x*, *y*)	(0.3250, 0.2694)	(0.4042, 0.4137)	(0.3867, 0.3862)
Concentration	0.1–2 mg/dL		
Total points	16,900		
Analysis points	13,528		
R(*R*^2^)	0.2555	0.8155	0.8522
G(R^2^)	0.7809	0.8984	0.9313
B(*R*^2^)	0.0738	0.3064	0.2877

D_2_ lamp: A deuterium arc lamp; H_2_ lamp: A halogen lamp; Mini- LEDs: Mini-light-emitting diodes; *R*^2^: determination coefficient; G: G grayscale values

Overall, noninvasive direct bilirubin detection by spectral analysis of color images using Mini-LEDs as the light source had the best linear relationship.

## Conclusions

This study proposes labeling bilirubin by redox, capturing images of test papers with a smartphone, and then performing RGB analysis using MATLAB image processing to quantify the color to achieve noninvasive detection of direct bilirubin using test papers. In this process, bilirubin is reduced to biliverdin by FeCl_3_ causing green color to appear on the test papers. When a D2 lamp, H2 lamp, and mini-LEDs were used as the light source, the color composition of the analyzed image was represented by its average grayscale value for three different channels (R, G, and B). The experimental results showed that for the direct bilirubin concentration range of 0.1–2 mg/dL, the green channel had the highest *R*^2^ values of 0.7809, 0.8984, and 0.9313, for the D2 lamp, H2 lamp, and mini-LEDs, respectively. Direct bilirubin concentrations higher than 0.56 mg/dL were detected, and a direct bilirubin concentration higher than 1.86 mg/dL could be quantitatively analyzed. The measured data showed that this method can more accurately analyze changes in a patient's direct bilirubin index and provide a reference for hospitals to successfully conduct telemedicine.

## Data Availability

The data presented in this study are available on request from the all authors.

## Abbreviations

FeCl_3_: Ferric chloride
Mini-LEDs: Mini-light-emitting diodes
LOD: Limit of detection
R: Red
G: Green
B: Blue
*R*^2^: Coefficient of determination coefficient
BOD: Bilirubin oxidase
GrONPs: Graphene oxide nanoparticles
CHIT: Chitosan
H2: Halogen
D2: Deuterium
CoA: Certificate of analysis
SDS: Safety data sheet
